# Pelvic injuries in elderly trauma patients: epidemiology, injury characteristics, in-hospital care and factors influencing mortality

**DOI:** 10.1007/s00402-026-06506-x

**Published:** 2026-09-10

**Authors:** Anders Enocson, Andrea Lundin, Mathias Granqvist

**Affiliations:** 1 Department of Trauma, Acute Surgery and Orthopaedics, Karolinska University Hospital, Stockholm, Sweden; 2 Department of Molecular Medicine and Surgery, Karolinska Institute, Stockholm, Sweden; 3 Department of Medicine, Karolinska Institute, Stockholm, Sweden

## Abstract

**Background:**

Pelvic and acetabular fractures are increasing in incidence in elderly patients. Whether the fracture contributes independently to mortality, or merely accompanies the factors that drive it, remains contested, and large national data on elderly trauma patients are limited. The primary aim of this study was to determine factors influencing 30-day mortality in elderly trauma patients, with particular focus on pelvic injuries. Secondary aims included to analyse the influence of these injuries on in-hospital care and discharge destination.

**Methods:**

All patients aged ≥ 60 years admitted as trauma alerts in Sweden between 1 January 2022 and 31 December 2024 were identified through the Swedish Trauma Registry (SweTrau). The primary outcome was 30-day mortality. The association between pelvic injury (pelvic or acetabular fracture) and 30-day mortality was assessed using multivariable logistic regression, adjusting for gender, ASA class, NISS, and head injury.

**Results:**

Of 11,624 patients (median age 76 years, 39.7% males), 776 (6.7%) sustained a pelvic injury. Patients with pelvic injury more frequently sustained high-energy trauma, had a higher median NISS (17 vs. 11), longer hospital length of stay (6 vs. 3 days), higher ICU admission rate (30.9% vs. 20.0%), and was less frequent discharged direct home (34.0% vs. 55.8%; all *p* < 0.001). Crude 30-day mortality was 16.1% vs. 12.5% (*p* = 0.002) for patients with and without pelvic injury respectively. After adjustment for gender, ASA class, NISS, and head injury, pelvic injury remained independently associated with 30-day mortality (adjusted OR 1.28, 95% CI 1.03–1.57, *p* = 0.025).

**Conclusion:**

In a national cohort of elderly trauma patients, pelvic injury was independently associated with increased 30-day mortality, greater in-hospital resource use and greater need for in-patient rehab after discharge, and should be considered a distinct prognostic factor in early risk stratification.

Keywords: pelvic injury, trauma, elderly, mortality, retrospective

## Background

Pelvic injuries (pelvic and acetabular fractures) have been increasing in incidence the past two decades in elderly patients and constitute an increasing burden on healthcare systems [1, 2]. In the elderly, a pelvic injury may represent either a marker of trauma severity (high-energy trauma), then frequently accompanied by haemorrhage and concomitant injuries, or a marker of frailty and osteoporosis (low-energy trauma). Although somewhat divergent, reported mortality in these patients is substantial; one-year mortality in elderly patients with pelvic or acetabular fractures has been reported between 16 and 33% [2–6]. Whether the fracture contributes independently to mortality, or merely accompanies the factors that drive it, remains contested. Some large trauma cohorts have identified pelvic fracture as an independent predictor of death after adjustment for associated injuries and patient characteristics with odds ratios of 1.9 to 2.1 [7]. In contrast, in a prior Swedish single-centre study there was no independent association with 30-day mortality for adults, concluding that the fracture indicated injury severity rather than causing death [8]. The outcome of a patient with a single pelvic injury caused by a low-energy trauma most likely differs from the patient with a pelvic injury in connection with a trauma causing a trauma-alert defined by national trauma guidelines [9]. Existing evidence derives largely from single-centre series, and large national population-level data on elderly trauma patients are limited. This is especially true for elderly patients involved in major trauma. The primary aim of the present study therefore was to determine factors influencing 30-day mortality in trauma patients aged ≥ 60 years with pelvic or acetabular fractures within the context of a large national cohort of trauma patients. Secondary aims included analyses of epidemiology, injury characteristics and in-hospital care.

## Methods

### Study population

Patients were identified through the Swedish Trauma Registry (SweTrau), a national registry that include all patients admitted as trauma alerts across Sweden. Variables are registered according to the Utstein template [10]. The registry has been reported to have high accuracy, correctness, and completeness [11]. Criteria for trauma alerts are nationally defined [9]. All patients aged ≥ 60 years admitted through trauma alert between Jan 1 2022 and Dec 31 2024 were eligible for inclusion.

Extracted variables from the registry comprised age, gender, American Society of Anaesthesiologists (ASA) class, injury mechanism, injury type, Injury Severity Score (ISS), New Injury Severity Score (NISS), concomitant injuries based on the Abbreviated Injury Scale (AIS), date of death, and in-hospital care data (hospital length of stay, intensive care, discharge destination). Pelvic injuries (pelvic and acetabular fractures) were identified and subclassified through ICD-10 codes. Comorbidity burden was approximated through ASA classification. Patients were excluded if they were declared dead on arrival, had no registered ICD-10 codes/injuries, or represented duplicate registry entries.

### Statistical analysis and outcomes

The primary outcome was 30-day mortality, compared between patients with and without pelvic injuries. Baseline characteristics were compared using the Mann-Whitney test for continuous variables and the χ² test for categorical variables. Categorical data were presented using frequency counts and percentage distributions, and continuous data were presented as medians with interquartile ranges (IQRs). The association between pelvic injury and 30-day mortality was assessed using logistic regression with stepwise addition of confounders; gender, ASA class (1–2 or 3–4), NISS (< 15 or ≥ 15), head injury (yes or no), and pelvic injury (yes or no). Pelvic injury was also assessed as an independent factor for hospital length of stay (dichotomized by ≥ 4 days (median for whole cohort)), ICU admission (yes or no) and discharge destination home (yes or no), adjusting for the same confounders. Results were presented as odds ratios (ORs) with 95% confidence intervals (95% CIs). Univariate comparisons were reported alongside the adjusted estimates. A two-sided p-value < 0.05 was considered statistically significant. All statistical analyses were performed using R version 4.2.3 (R Foundation for Statistical Computing, Vienna, Austria).

## Results

11,624 patients were included, of which 776 patients had a pelvic injury (pelvic or acetabular fracture). Median age for all patients was 76 (IQR 67–83.3) years with 39.7% males, equally distributed between ASA 1–2 and 3–4 groups (49.5% and 50.5% respectively), and with 37.7% arriving with a NISS ≥ 15 (Table 1).

### Patient characteristics and injury mechanisms

Among the 776 patients with a pelvic injury, median age was 76 years (IQR 68–84) and 348 (44.8%) were men, compared with 76 years (IQR 67–83) and 4269 (39.4%) men among the 10,848 patients without pelvic injury (Table 1). Comorbidity burden, approximated by ASA classification, was lower in the pelvic injury group, with 442 (57.3%) classified as ASA 1–2 compared with 5292 (49.0%) of those without (*p* < 0.001). Injury mechanism distributions differed substantially between groups. Patients with pelvic injuries had more frequently sustained high-energy trauma, including high-energy falls (34.4% vs. 23.5%), motor vehicle accidents (13.3% vs. 9.7%), pedestrian injuries (8.9% vs. 2.2%) and motorcycle accidents (4.7% vs. 3.0%). Conversely, low-energy falls accounted for a smaller proportion in the pelvic injury group (25.5% vs. 46.1%). Almost all pelvic injuries resulted from blunt trauma (99.4%).

### Injury severity, in-hospital care and discharge destination

Patients with pelvic injuries had a higher overall injury burden, with a median NISS of 17 (IQR 12–27) compared with 11 (IQR 4–19) in patients without (*p* < 0.001). In addition, 444 (57.2%) had a NISS ≥ 15 compared with 3,936 (36.3%) (*p* < 0.001). Despite a higher overall injury severity, head injury was less frequent in the pelvic injury group (36.9% vs. 47.2%, *p* < 0.001). Median hospital length of stay was longer for patients with pelvic or acetabular fracture (6 days, IQR 3–12) than for those without (3 days, IQR 2–7) (*p* < 0.001). Patients with pelvic injury were admitted to the ICU in higher rates compared to those without (30.9% vs. 20.0%, *p* < 0.001). Pelvic injury was also associated with being discharged directly home less often (34% vs. 55.8%, *p* < 0.001). After adjustment for gender, ASA class, NISS and head injury, pelvic injury was independently associated with hospital length of stay ≥ 4 days (adjusted OR 1.87, 95% CI 1.59–2.19), ICU admission (adjusted OR 1.33, 95% CI 1.11–1.58), and reduced odds of direct discharge home (adjusted OR 0.45, 95% CI 0.38–0.53); all *p* < 0.001.

### Pelvic and acetabular fractures

Of the 776 patients with pelvic injury, 700 sustained a pelvic ring fracture and 193 an acetabular fracture, with a subset of patients sustaining both. Acetabular fractures showed a clear male predominance (132 males vs. 61 females, *p* < 0.001), whereas pelvic ring fractures were more evenly distributed by gender, with a smaller male excess (375 males vs. 325 females, *p* = 0.007). Thirty-day mortality did not differ significantly by gender within either fracture subtype (Table 2).

### Mortality

Overall 30-day mortality in the cohort was 12.7% (1458/11624). Crude 30-day mortality was higher in patients with pelvic injuries (16.1%, *n* = 125) than in those without (12.5%, *n* = 1353) (*p* = 0.002). In univariable logistic regression, pelvic injury was associated with an OR of 1.35 (95% CI 1.10–1.64, *p* = 0.002) for 30-day mortality. After adjustment for gender, ASA class, NISS and head injury, the association persisted, with an adjusted OR of 1.28 (95% CI 1.03–1.57, *p* = 0.025) (Table 3).

**Table 1:** Patient and injury characteristics

Variable	All patients (*n* = 11624)	Patients with pelvic injury (*n* = 776)	Patients without pelvic injury (*n* = 10848)
Age (years), median (IQR)	76 (67–83)	76 (68–84)	76 (67–83)
Gender, male (%)	4617 (39.7)	348 (44.8)	4269 (39.4)
ASA class 1–2 (%)	5734 (49.5)	442 (57.3)	5292 (49.0)
ASA class 3–4 (%)	5844 (50.5)	329 (42.7)	5515 (51.0)
Injury mechanism, n (%)			
MVA	1150 (10.0)	103 (13.3)	1047 (9.7)
MCA	355 (3.1)	36 (4.7)	319 (3.0)
Bicycle accident	790 (6.8)	64 (8.3)	726 (6.7)
Pedestrian	306 (2.7)	69 (8.9)	237 (2.2)
Traffic other	96 (0.8)	6 (0.8)	90 (0.8)
Shot	26 (0.2)	3 (0.4)	23 (0.2)
Stabbed	260 (2.3)	0 (0)	260 (2.4)
Struck by blunt object	383 (3.3)	29 (3.7)	354 (3.3)
Low energy fall	5161 (44.7)	197 (25.5)	4964 (46.1)
High energy fall	2799 (24.3)	266 (34.4)	2533 (23.5)
Blast injury	15 (0.1)	0 (0)	15 (0.1)
Other	195 (1.7)	1 (0.1)	194 (1.8)
Type of injury, n (%)			
Blunt	11,287 (97.3)	771 (99.4)	10,516 (97.1)
Penetrating	315 (2.7)	5 (0.6)	310 (2.9)
NISS, median (IQR)	11 (4–20)	17 (12–27)	11 (4–19)
NISS ≥ 15, n (%)	4380 (37.7)	444 (57.2	3936 (36.3)
Head injury, n (%)	5408 (46.5)	286 (36.9)	5122 (47.2)

*ASA* american society of anesthesiologists, *IQR* interquartile range, *MCA* motorcycle accident, *MVA* motor vehicle accident, *NISS* new injury severity scale, *SBP* systolic blood pressure, *Shock* SBP < 90 mmHg

**Table 2:** Type of pelvic injury in relation to gender and 30-day mortality

Injury type	All (*n* = 776)	Gender			30-day mortality		
		Male (*n* = 428)	Female (*n* = 348)	*p*-value	Male (*n* = 428)	Female (*n* = 348)	*p*-value
Acetabulum	193	132	61	0.02	17	5	0.48
Pelvic	691	369	322	< 0.001	54	55	0.44

**Table 3:** Logistic regression to assess risk factors for 30-day mortality

	30-day mortality	Univariable		Multivariable	
	*n* (%)	OR (95% CI)	*p*-value	OR (95% CI)	*p*-value
Gender					
Male	895 (12.77)	*Ref*		*Ref*	
Female	583 (12.62)	0.99 (0.88–1.10)	0.818	1.09 (0.97–1.23)	0.140
ASA class					
1–2	432 (7.53)	*Ref*		*Ref*	
3–4	1033 (17.68)	2.64 (2.34–2.97)	< 0.001	2.69 (2.38–3.04)	< 0.001
NISS					
< 15	489 (6.75)	*Ref*		*Ref*	
≥ 15	989 (22.58)	4.03 (3.59–4.53)	< 0.001	3.60 (3.19–4.07)	< 0.001
Head injury					
No	505 (8.12)	*Ref*		*Ref*	
Yes	973 (18.00)	2.48 (2.21–2.78)	< 0.001	1.83 (1.62–2.07)	< 0.001
Pelvic injury					
No	1353 (12.47)	*Ref*		*Ref*	
Yes	125 (16.11)	1.35 (1.10–1.64)	0.003	1.28 (1.03–1.57)	0.025

*ASA* american society of anesthesiologists, *CI* confidence interval, *NISS* new injury severity score, *OR* odds ratio

## Discussion

In this national cohort of 11,624 trauma patients aged ≥ 60 years, the main finding was that a pelvic injury was identified as an independent risk factor for 30-day mortality after adjusting for several other relevant trauma variables. The pelvic injury therefore appears to contribute independently to short-term mortality in the elderly trauma population, beyond what is explained by overall injury severity, physiological reserve, or concomitant head injury.

This finding contrasts with the earlier single-centre Swedish study by Holtenius et al., which used SweTrau data from Karolinska University Hospital between 2013 and 2015 and found no independent association between pelvic fracture and 30-day mortality once age, ISS, ASA, GCS, and shock were accounted for [8]. Several factors may explain the divergence. Our study is national rather than single-centre, restricted to patients aged ≥ 60 years, and substantially larger, providing greater statistical power to detect a modest but clinically relevant effect. The age cut off at ≥ 60 years represents a widely adopted convention in trauma research and is frequently employed in studies assessing outcomes and survival and is a key difference [12–14]. The magnitude of effect we observed is nonetheless smaller than that reported in the larger US blunt-trauma cohort by Schulman et al., in which pelvic ring fracture conferred an adjusted odds ratio of approximately 1.9–2.1 for in-hospital mortality across all adult ages [7]. Our point estimate sits between these two prior reports and is consistent with the broader literature suggesting that pelvic and acetabular fractures, while strongly correlated with injury severity, retain a measurable independent contribution to mortality in elderly patients [4, 15–18].

The clinical interpretation of this association is shaped by the bimodal injury mechanism in elderly trauma. In our cohort, patients with pelvic injuries more frequently sustained high-energy trauma, with a median NISS of 17, compared with 11 in those without, suggesting that part of the excess mortality reflects associated injuries and haemorrhagic risk inherent to high-energy mechanisms. At the same time, low-energy falls still accounted for a quarter of the pelvic injuries, and in this subset the fracture is more plausibly a marker of frailty and reduced physiological reserve, analogous to a fragility hip fracture. The substantial long-term mortality reported in geriatric pelvic ring fracture cohorts supports this dual interpretation. Lenz and colleagues reported a five-year mortality of 62.1% in geriatric patients (mean age 79 years, 67% with ASA class 3 or above), and a recent systematic review and meta-analysis of fragility pelvic fractures reported similarly high mortality estimates [2, 5]. Frailty has been independently associated with worse short-term outcomes after low-energy pelvic fracture in elderly adults, and the present adjusted OR of 1.28 likely captures the residual contribution of both pathways after accounting for NISS and ASA — the fracture appears to add prognostic information beyond what these summary measures convey [19].

These findings have implications for early triage and risk stratification of elderly trauma patients. The addition of a single anatomical fracture category meaningfully shifts mortality risk beyond what is captured by global injury severity scoring, supporting explicit consideration of pelvic or acetabular fractures as a distinct prognostic factor in this population rather than relying solely on NISS or ASA class. Whether this should influence acute management, for example, threshold for damage-control resuscitation, angioembolisation, or admission to higher-acute-care levels, cannot be answered by the present data, but the consistency of an independent mortality signal across geographically and methodologically diverse cohorts argues for that the fracture is more than an incidental finding [4, 7, 15–18]. Management decisions in the low-energy subgroup raise distinct questions. In cases were surgical treatment is indicated, early definitive fixation of pelvic and acetabular fractures are normally desirable [20]. Early operative fixation in fragility pelvic fractures has been associated with higher mortality and complication rates at one year in patients who cannot be mobilised within three to five days, highlighting the need for individualised assessment rather than uniform algorithms in the elderly [21].

Furthermore, we found that a head injury was an independent risk factor for mortality in this elderly trauma population. This finding was no surprise, and head injury remain the number one killer in severely injured polytrauma cases in conjunction with blunt trauma in most published series [22].

Patients with pelvic injury required more in-patient resources than those without, with a longer median hospital length of stay, a higher proportion admitted to intensive care, and more frequent need for onward referral at discharge. These findings corroborate those of Garcia et al., whose cohort of elderly patients with severe pelvic injury had a median hospital length of stay of 6 days, though with an ICU admission rate of 46%, compared to our 30.9% [15]. Klüter et al., who also included pelvic injury severity observed that this was proportional to hospital length of stay [23]. These findings indicate that the impact of pelvic and acetabular fracture in elderly trauma patients extends beyond short-term mortality to substantial demands on in-patient and post-acute care, which warrants attention in service planning and may be a useful complementary outcome in future studies.

### Limitations

This study has several limitations. The analysis was retrospective and observational, and residual confounding cannot be excluded. Measures of frailty aside from ASA classification and surgical complications could not be controlled for given the parameters available in the registry. Injury mechanism was not stratified in the primary model, conflating high- and low-energy injuries that likely differ biologically and prognostically; subgroup analyses by mechanism would strengthen causal inference but were beyond the scope of the present study. Fracture classification was based on ICD-10 coding rather than radiographic review, precluding analysis and classification by Judet, Tile or Young–Burgess fracture subtype, by displacement, or by whether the fracture was open or closed. Operative management, including modality and timing of fixation, was not captured; we cannot therefore assess whether intervention modified mortality risk, although prior data suggest that surgery within 72 h does not reduce mortality in fragility fractures [3]. The primary outcome was restricted to 30-day mortality, which does not capture longer-term mortality or functional recovery, both of which are highly relevant in this population [2, 17, 18].

## Conclusion

In this national cohort of trauma patients aged 60 years or older, pelvic injury was independently associated with increased 30-day mortality after adjustment for gender, ASA class, NISS, and head injury. In addition, these patients had longer hospital length of stay, increased need of intensive care and were less often discharged direct to their home. This supports consideration of pelvic or acetabular fracture as a distinct prognostic factor in early risk stratification of elderly trauma patients.

## Data Availability

Patient and mortality data that support the findings of this study are available from Svenska Traumaregistret (SweTrau), but restrictions apply to the availability of these data, which were used under licence for the current study and so are not publicly available. The data are, however, available upon request and with the permission of Svenska Traumaregistret (SweTrau).
